# Combining BulkFormer and TabPFN to predict post-transplant function from kidney biopsies during normothermic machine perfusion or cold storage

**DOI:** 10.1038/s41598-026-68541-3

**Published:** 2026-08-27

**Authors:** Samuel J. Tingle, Georgios Kourounis, Sofia Kazerouni, Harry V. M. Spiers, Miguel Larraz, Maithili Mehta, Serena MacMillan, Sarah A. Hosgood, Michael L. Nicholson, Neil S. Sheerin, Colin H. Wilson

**Affiliations:** 1 Translational and Clinical Research Institute, Newcastle University, Newcastle upon Tyne, UK; 2 National Institute of Health and Care Research Blood and Transplant Research Unit, Newcastle University and Cambridge University, Newcastle upon Tyne, UK; 3 Institute of Transplantation, Freeman Hospital, Newcastle upon Tyne, UK; 4 Department of Transplantation, Addenbrooke’s Hospital, Cambridge, UK; 5 Department of Surgery, University of Cambridge, Cambridge, UK

**Keywords:** BulkFormer, TabPFN, Foundation model, Transcriptomics, Bulk RNA-Seq, Kidney transplantation, Biological techniques, Biomarkers, Computational biology and bioinformatics, Nephrology

## Abstract

Generating predictions from transcriptomic data poses unique challenges due to the high number of genes, and often small sample size. BulkFormer and TabPFN have emerged as leading transformer-based foundation models for bulk transcriptomic and tabular data respectively. We explore an artificial intelligence pipeline using BulkFormer-TabPFN v2.5 which generates zero-shot predictions from RNA-Seq count data without retraining. This was tested on three cohorts of biopsies taken from donated human kidneys. BulkFormer-TabPFN could predict delayed kidney function using RNA-Seq counts from kidneys undergoing ex-situ normothermic machine perfusion (NMP; c-statistic = 0.82, 95% CI 0.67–0.97). Predictive discrimination was optimised under the following conditions: BulkFormer-TabPFN versus TabPFN alone, maximum absolute aggregation of BulkFormer gene-level embeddings. Cold storage biopsies showed poor predictive performance. BulkFormer-TabPFN predictions were modified by cytokine filter treatment during ex-situ NMP, suggesting they could be a dynamic surrogate endpoint for novel therapeutics, which is intrinsically linked to post-transplant outcome. This demonstrates for the first time synergistic benefits of these foundation models, to generate zero-shot predictions without model retraining. Although this work is exploratory, the provided code provides a blueprint to replicate generating predictions from RNA-Seq count data, which could be applied in a wide range of biomedical contexts within and beyond organ transplantation.

**Supplementary Information:**

The online version contains supplementary material available at https://doi.org/10.1038/s41598-026-68541-3.

## Introduction

Lack of certainty around how a donated organ will function after transplantation frequently leads to organ non-use, contributing to a global shortage of transplants^1^. A technique termed normothermic machine perfusion (NMP), where a warm blood-based solution is circulated through an isolated organ to maintain it outside of the body, provides an opportunity to assess organ health^2–4^. However, more accurate measures of kidney viability during NMP are urgently needed^4,5^. Previous studies have identified transcriptomic signatures during kidney NMP that are associated with poor graft outcomes post-transplant, highlighting the potential for using transcriptomic analysis in this setting^6,7^.

However, using transcriptomic data for prediction is challenging for several reasons. Firstly, data is generally available for relatively few samples, compared to the large clinical cohorts underpinning most prediction models^8^. Secondly, there is a very large number of features, with information on tens of thousands of gene transcripts per sample. Standard statistical prediction modelling has to reduce this large number of genes to select a set of promising genes to use for modelling; a process where overfitting and overoptimism are virtually guaranteed, especially in small datasets^9–11^. In addition, this manual process is inherently variable and analyst-dependent, introducing potential bias in feature selection and reducing reproducibility.

Combining BulkFormer and TabPFN v2.5 may allow us to overcome these issues^12–15^. These are both state of the art foundation models which use a transformer-based architecture; the same architecture type underpinning large language models such as ChatGPT. The models take in data as a ‘prompt’, and output distilled information or predictions, without requiring any model retraining; this is termed “zero-shot” predictions. TabPFN has been at the heart of a recent revolution in the analysis of tabular data (data organised in rows and columns)^16^. It is a state of the art model, outperforming established machine learning approaches for tabular data, such as XGBoost and random forests^13^. TabPFN is especially strong when sample size is low, making it an attractive option for use with current transcriptomic datasets. However, performance has been reported to degrade with high-dimensional data, especially when the number of features exceeds 2000^13,17^.

Standard approaches to dimensionality reduction often lose important data, or rely on relationships between predictors and outcome thereby potentially amplifying noise leading to overfitting. BulkFormer is a foundation model trained on over 500,000 bulk transcriptomic samples^14,15^. It can process transcriptomic data on 20,010 genes and distil this information into a compact set of embeddings, each of which reflects a key biological programme. This distilled data can then be used for a range of tasks, including disease annotation, patient prognosis prediction and drug response prediction^14,15^.

Despite these conceptual advantages to deal with the specific challenges posed by transcriptomics data sets, there are no published articles describing the combination of BulkFormer and TabPFN v2.5. Our aim was therefore to evaluate the performance of a novel combination BulkFormer-TabPFN pipeline to make outcome predictions based on raw transcriptomic count data.

## Methods

**Fig. 1:**
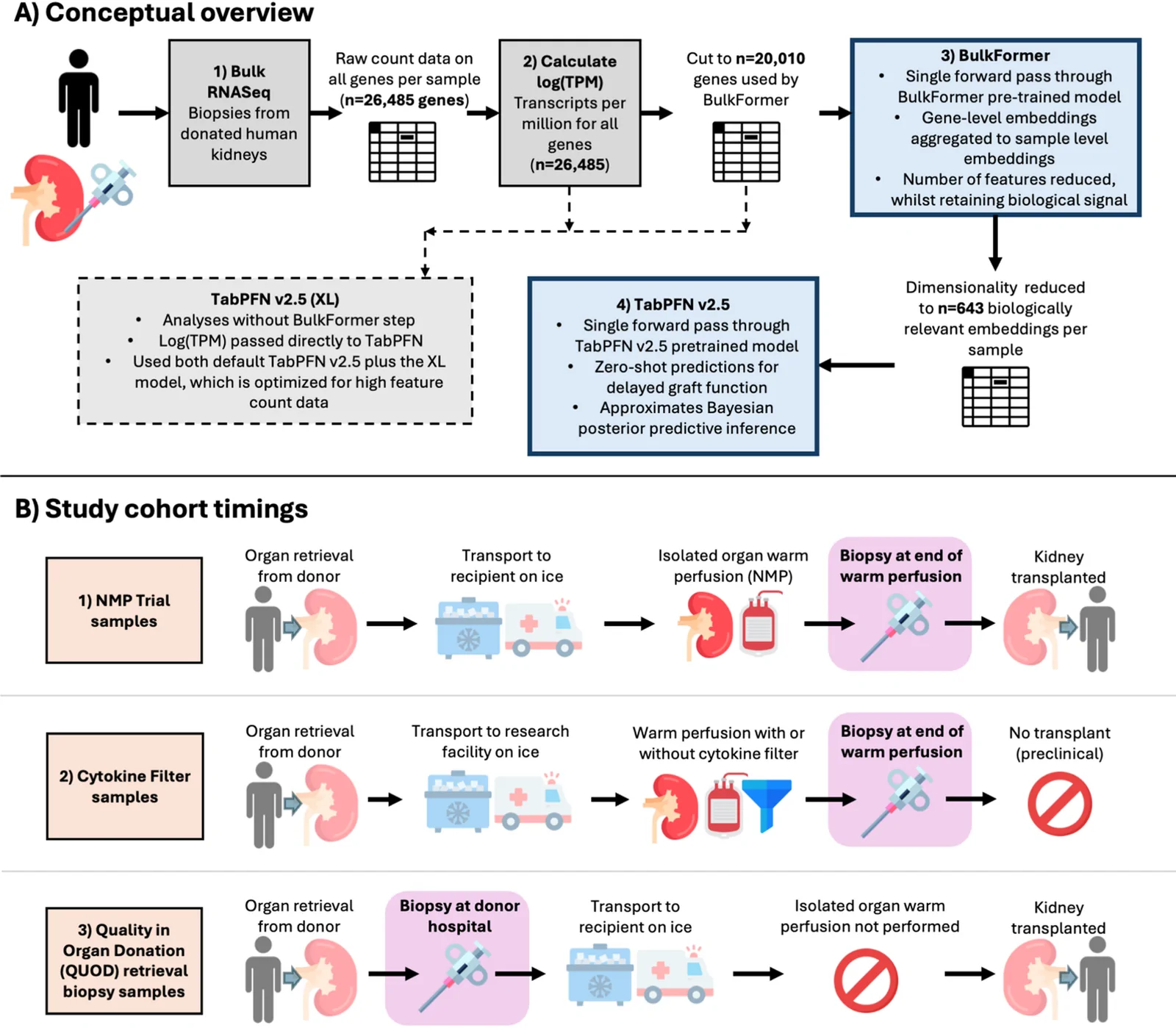
Study overview. (**A**) Conceptual overview of computational pipeline to generate predictions on post-transplant outcome from bulk RNA-Seq raw count data. (**B**) Overview of the three study cohorts, showing timing of kidney biopsy, relative to other key pretransplant events.

This is an exploratory study to assess the ability of a BulkFormer-TabPFN pipeline to generate clinical outcome predictions from small sample-size transcriptomic datasets. All data and code used in this study is available in our accompanying GitHub and Zenodo (https://doi.org/10.5281/zenodo.21332770) repositories^18,19^. Ethical approval was not required as all raw data (RNA-Seq raw counts and metadata) was retrieved from previous publications, either via the gene expression omnibus (GEO) or direct contact with the study authors. This raw count data, along with its metadata, is also available in our GitHub repository^18^. A summary of the computing pipeline is given in Fig. 1A. All computing steps shown were performed zero-shot, without any model retraining.

Our primary clinical outcome was delayed graft function (DGF), often defined as the need for dialysis within the first week post-transplant. This measure of early post-transplant kidney function has been shown to correlate with long-term kidney survival, and is frequently used as the primary outcome in both preclinical and clinical research^5,6,20,21^.

As dialysis in the first day post-transplant often reflects hyperkalaemia, rather than truly reflecting function of the transplanted kidney, recipients receiving dialysis only during the first 24 h post-transplant (with no further dialysis in the first week), were classified as not having DGF. Therefore, our definition of DGF was requirement for dialysis in the first week post-transplant, outside of the first 24 h. This approach is consistent with prior work demonstrating that transplants where dialysis is only performed in the first 24 h have transcriptomic profiles comparable to kidneys where no post-transplant dialysis is performed^6,7^.

### Summary of study cohorts and methodology

A summary of the three study cohorts is given in Fig. 1B; Table 1. All three cohorts include bulk RNA-Seq raw count data from renal cortex tissue biopsies of human kidneys donated for transplantation. For all cohorts, tissue was homogenised, and RNA isolated with the pure link RNA mini kit (Ambion). Library preparation was performed using the TruSeq Stranded total RNA library prep kit (Illumina), and sequenced on a Hiseq sequencer (Illumina). Alignment to the human genome (Hg38) was performed with HISAT2, and featurecounts was used to generate the raw count data^6,20^. Further details on the study cohorts, and on precise methodologies for generating the raw count data, can be found in the original publications^6,20^.

**Table 1:** Study cohort details, and our aims for each cohort. Normothermic Machine Perfusion (NMP) is a way of keeping an isolated kidney alive outside of the body. Delayed graft function (DGF) was our primary measure of how well the kidney was functioning after transplant.

Cohort	Details	Source	Aim
NMP trial samples	*N* = 34 kidneys biopsied during the United Kingdom NMP randomised trial^5^ All kidneys were subsequently transplanted, with data on early post-transplant function available for all. 9/34 recipients experienced DGF	Data provided upon request from the authors of Ferdinand 2021^6^	Assess ability of our pipeline to predict DGF using biopsies taken during NMP
Cytokine filter samples	*N* = 10 kidneys (5 pairs). Preclinical normothermic machine perfusion with or without a cytokine filter treatment. The aim of the cytokine filter treatment is to improve post-transplant kidney function	Ferdinand 2021^6^ and associated GEO accession GSE121447	Assess whether DGF predictions are modifiable by therapies delivered during NMP
QUOD retrieval biopsy samples	Kidneys biopsied at the donor hospital (before transport of the kidney on ice). These biopsies were collected and stored in the Quality in Organ Donation biobank. *N* = 263 kidneys with sufficient data on DGF. 73/263 recipients experienced DGF	Zhang 2024^20^ and associated GEO accession GSE215108	Assess ability to predict DGF with retrieval biopsies (in kidneys not receiving NMP)

The NMP trial cohort consists of biopsies performed at 1 h of NMP as part of the United Kingdom NMP randomised trial^5^. The data for *N* = 34 kidneys was kindly provided by Ferdinand et al., and included the 33 kidneys included in their manuscript, plus an additional case. All biopsies had linked DGF data, in-line with our definition above^6^.

The Cytokine Filter cohort included paired human kidney experiments (*N* = 10 kidneys). These were performed on human kidneys retrieved for transplant but then deemed unsuitable and offered for research. One kidney per pair received 4 h of NMP plus cytokine filter treatment, and the other kidney was an NMP-only control. None of these kidneys were transplanted. Raw count data was taken from GEO (accession GSE121447), with additional metadata provided in the associated manuscript^6^. This cohort allowed us to assess whether DGF predictions were modifiable by therapeutic intervention during NMP, with the paired design being a particular strength^6^.

The Quality in Organ Donation (QUOD) retrieval biopsy cohort contains biopsies which were taken at the time of kidney organ retrieval (following cold flush of the organs), prior to static cold storage and transport^20^. Raw count data was taken from GEO (accession GSE215108), with metadata taken from manuscript supplementary data^20^. Of the available biopsies, 263 had sufficient metadata to allow labelling of DGF in line with our definition above.

The NMP and QUOD retrieval biopsy cohorts were analysed and evaluated entirely independently, with no cross-cohort prediction between NMP and QUOD retrieval biopsy cohorts.

### Processing of raw count data

The raw count data for all three cohorts was indexed using gene symbols, rather than the Ensembl IDs required for BulkFormer. These gene symbols were mapped to Ensembl gene IDs using Ensembl BioMart (using biomaRt; *hsapiens_gene_ensembl*)^22–24^. Symbols not mapped by BioMart were resolved via the HGNC REST API to identify approved symbols from aliases/previous symbols, and these approved symbols were then re-mapped to Ensembl gene IDs via biomaRt^24,25^. The mapping file was created on 11th Feb 2026 using the most recent Ensembl (release 115) and HGNC versions, and is available on our GitHub^18,22^.

Where a single gene symbol mapped to multiple Ensembl IDs, we prioritised Ensembl IDs that are used in the BulkFormer pipeline (either as one of the input genes or a gene with length information for their transcript per million, TPM, calculation)^26,27^. Counts for any duplicate Ensembl ID were summed. Any gene symbols which could not be mapped to Ensembl ID used by BulkFormer were initially retained for TPM calculations. Raw count data was then converted to TPM and log-transformed to log_e_(TPM); this was performed using code provided in the BulkFormer GitHub, to ensure consistency with the data used for previous BulkFormer training^26^.

### Generating BulkFormer embeddings

We used the BulkFormer (version 2) 147 M model to generate embeddings^14,15,26,27^. The BulkFormer GitHub repository was cloned on 10th January 2026, and all associated files (including model weights and gene annotation files) were downloaded on the same day from Zenodo^27^. The code for generating embeddings was taken directly from the “bulkformer_extract_feature.ipynb” Jupyter notebook.

The key area where the BulkFormer code was modified was the method for aggregating gene-level to sample level embeddings. BulkFormer generates latent space embeddings (*n* = 643 for the model used here) per gene, per sample. For prediction tasks, gene-level embeddings need to be aggregated to sample-level embeddings, such that each sample is now represented by *n* = 643 unique embeddings. The available options for aggregation were: maximum (used in BulkFormer manuscript)^14^, mean and median. We have added an option for maximum absolute value, so that large negative embeddings are also captured. As sensitivity analyses we also added options for using the 99.9th percentile rather than the maximum (attempting to make the maximum aggregation approaches robust to potential single gene outliers). These new options are available on our GitHub^18^.

### TabPFN v2.5

TabPFN v2.5 was used with model weights downloaded from Hugging Face (a repository for model weights) on 19th February 2026^28^. We utilized the default model (tabpfn-v2.5-classifier-v2.5_default.ckpt) for all feature sets and additionally evaluated the XL model variant (tabpfn-v2.5-classifier-v2.5_large-features-XL.ckpt) for log(TPM) data. The models operate by generating predictions conditioned on contextual examples from the input data, rather than undergoing traditional parameter retraining.

The input to these models was either: (1) BulkFormer sample-level embeddings (*n* = 643 features) generated as described above, and (2) log(TPM) values containing either all genes or only the 20,010 genes used for BulkFormer inference; this was done to assess the impact of omitting the BulkFormer step.

We evaluated model performance using leave-one-out cross-validation (LOO-CV) within the NMP trial and QUOD retrieval biopsy cohorts separately. Within each cohort, outcome-labelled data were provided to the pipeline as context, with an outcome value for a single kidney (occurrence of DGF) masked and predicted by the model. This process of masking a single kidney’s outcome was repeated for all kidneys within the cohort, allowing comparison of predicted and observed outcomes to assess predictive performance. This was performed in the NMP trial and QUOD cohorts completely separately (neither cohort used any information from the other cohort in any way).

We also generated the predicted probability of DGF for each sample in the cytokine filter cohort, using the NMP trial cohort as context. This was performed to assess whether DGF predictions were dynamic, and whether they could be influenced by novel therapies. We refer to these as “pseudoprobabilities” because these biopsies were taken at different timepoints of NMP compared with the NMP trial cohort. Therefore, they do not represent cohort-calibrated estimated true probabilities. Higher values indicate a greater predicted probability of DGF compared with other cases in the dataset, but the numerical values themselves should not be assumed to represent absolute probabilities.

To assess whether TabPFN predictions were strongly associated with individual genes or BulkFormer embeddings, associations between transcriptomic features and the TabPFN-predicted log-odds of delayed graft function were assessed. Gene-level associations were modelled from raw RNA-sequencing counts using DESeq2, with predicted log-odds included as a continuous explanatory variable^29^. Associations with individual BulkFormer embedding dimensions were assessed using limma linear models with empirical Bayes moderation (trend = TRUE, robust = TRUE)^30^. Benjamini–Hochberg adjustment was applied separately to each analysis. Results were visualised using volcano plots and heatmaps.

### Statistical analysis

Statistical analysis and reporting were conducted in line with recent best-practice guidance for reporting binary prediction model performance in medical AI research^31^. Model discrimination was assessed using the c-statistic (area under the receiver operating characteristic curve, AUC), which was prespecified as the primary performance metric. Overall predictive performance was evaluated using the Brier score^31^. Confidence intervals (CI) were calculated using a bootstrap approach for all metrics, except for the c-statistic, where the DeLong method implemented in the pROC R library was used^32^.

For visualisation, ROC curves were plotted to illustrate discrimination, and violin plots were used to display the distribution of performance estimates across feature representations and aggregation strategies. The code used for performance evaluation and visualisation was adapted from the best-practice guidance paper’s accompanying GitHub repository^33^. To assess the difference in pseudoprobabilities of DGF in the paired kidney experiments (Cytokine Filter cohort) we used a two-tailed paired t-test.

## Results

The novel computing pipeline is displayed in Fig. 1A, and a summary of study cohorts is given in Table 1; Fig. 1B. Kidney cohort sample sizes were: 34 for the NMP trial samples (9/34 with DGF), 10 for the Cytokine Filter preclinical cohort (5 pairs) and 263 for the QUOD retrieval biopsy cohort (73/263 with DGF). Full details on every tested model is given in Supplementary Table 1, and includes all predictive model metrics recommended by Calster et al.^31^.

### Combining BulkFormer and TabPFN allows DGF prediction

**Fig. 2:**
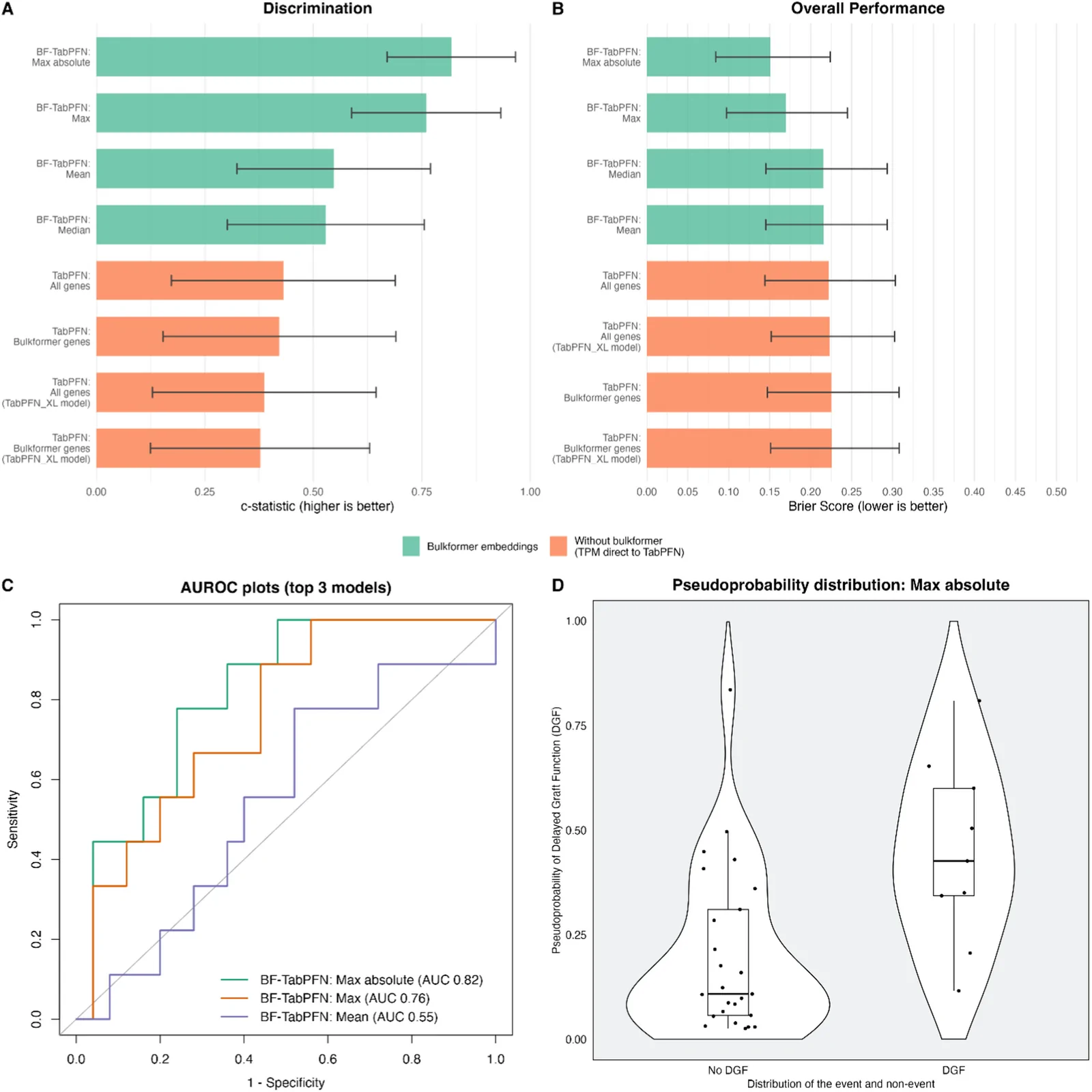
Predictive performance for early post-transplant kidney function (defined as Delayed Graft Function; DGF) in the NMP Trial cohort. The discrimination (measured using c-statistic) and overall performance (Brier score) for each of the tested models are shown in panel (**A**) and (**B**), and are ranked best to worst. Models using the full BulkFormer-TabPFN pipeline are shown in green, and are labelled with which BulkFormer method was used for aggregation of gene-level embeddings (e.g. Max absolute). Models using only TabPFN directly on the transcriptomic data (without BulkFormer) are shown in orange, and labelled with which genes were used, and whether the TabPFN default or high feature count (XL model) were used. Error bars represent 95% confidence intervals. (**C**) Receiver-operating characteristic curves for the top three performing models by c-statistic. (**D**) Distribution of predicted probabilities stratified by subsequent delayed graft function status.

We initially analysed the NMP trial cohort. Our BulkFormer-TabPFN pipeline allowed RNA-Seq raw count data from biopsies taken at 1 h of normothermic machine perfusion to be used to predict early post-transplant function, as measured by delayed graft function (Fig. 2).

Assessing various options for aggregation of BulkFormer gene-level to sample-level embeddings revealed that maximum absolute aggregation resulted in the best model performance (Fig. 2A,B). Maximum absolute and maximum aggregation produced models with C-statistics whose 95% confidence intervals excluded 0.5, indicating discrimination significantly better than chance, whereas mean and median aggregation did not. As a sensitivity analysis we also aggregated embeddings using 99.9th percentile, to see whether maximum embeddings were overly influenced by outliers; these models showed inferior predictive performance (Supplementary Table 1).

We next assessed predictive performance of a pipeline which did not use BulkFormer and instead applied TabPFN directly to the transcriptomic data (Fig. 1A). When the BulkFormer step was omitted, predictive performance was lost, with TabPFN-only pipelines generating predictions that were no better than chance (Fig. 2A,B). This was true for both the default TabPFN v2.5 models, and TabPFN models specifically trained for high feature count data (the v2.5 XL models).

Figure 2C displays receiver-operating characteristic curves for the top three performing model pipelines, based on c-statistic. The best overall model was the BulkFormer-TabPFN model which used maximum absolute embedding aggregation (c-statistic = 0.82, 95% CI 0.67–0.97; Brier score = 0.15, 95% CI  0.08–0.22). The distribution of predicted probabilities of DGF, stratified by actual DGF status, is displayed in Fig. 2D.

Overall, no individual gene or BulkFormer embedding showed a uniformly consistent association with TabPFN predictions or actual post-NMP outcome (Supplementary Fig. 1), suggesting that TabPFN predictions reflect complex multivariable patterns across the transcriptomic embeddings rather than reliance on a single dominant feature.

### Predicted DGF probability is dynamic and modulated by a novel therapy

To assess whether the predicted DGF probability from this pipeline is sensitive to therapeutic modulation of the kidney, we used a cohort of biopsies from a preclinical study of a novel therapy^6^. This consisted of paired kidneys (i.e. both kidneys from the same donor) undergoing normothermic machine perfusion with or without cytokine filter treatment.

**Fig. 3:**
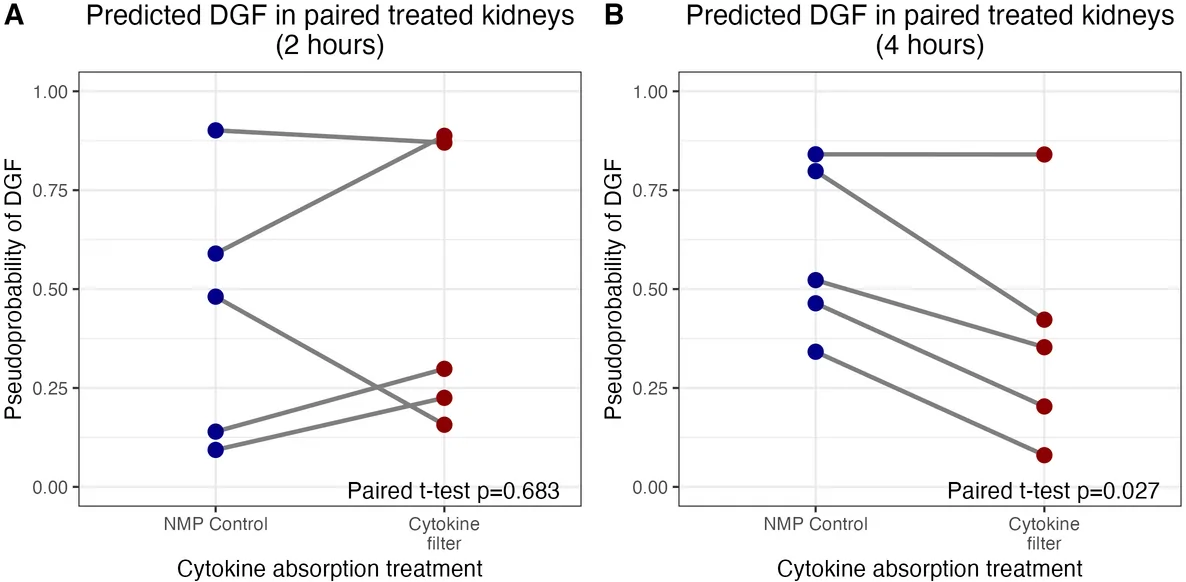
Predicted pseudoprobability of delayed graft function (DGF) in the Cytokine Filter cohort. Each dot is a single kidney, with lines connecting pairs of kidneys. The predictions were generated based on the NMP Trial samples, using the BulkFormer TabPFN pipeline with the maximum absolute aggregation method. Panels represent cortex biopsy samples taken at 2 h and 4 h of normothermic machine perfusion (**A** and **B** respectively). DGF, delayed graft function.

Using embedding and outcome data from the NMP trial cohort as the context, we generated zero-shot predicted probability of DGF for each of the Cytokine Filter kidney biopsies (displayed in Fig. 3). As the machine perfusion timepoints differed between groups (1 h in NMP trial cohort; 2 and 4 h in cytokine filter cohort), we have labelled this as a pseudoprobability. At the end of cytokine filter treatment (4 h), there was a significant reduction in predicted pseudoprobability of DGF in the Cytokine Filter group (mean difference in DGF pseudoprobability = − 21.3%, 95% CI − 38.7% to − 4.0%, paired t-test *p* = 0.027) compared to the control group. This occurred despite no difference between groups at the 2-hour timepoint (mean difference in DGF pseudoprobability = + 4.7%, 95% CI − 24.8% to 34.2%, *p* = 0.683). Figure 3 displays these results.

### Biopsy during NMP allows better predictions than biopsies during cold storage

We next assessed whether the same pipeline (Fig. 1A) could be used to predict DGF, in an independent cohort of biopsies taken from kidneys flushed with cold preservation solution (thus at 4 °C temperature) at the time of organ retrieval (QUOD cohort), as opposed to kidney biopsies taken during normothermic machine perfusion (Fig. 1B). This analysis was conducted entirely within the QUOD cohort, with no use of NMP trial samples at any stage, including as ‘context’. Full model performance metrics in the retrieval biopsy cohort are provided in Supplementary Fig. 2 and Supplementary Table 1.

Mirroring the results in the NMP cohort, the BulkFormer-TabPFN model incorporating maximum absolute embedding aggregation ranked highest by c-statistic (Supplementary Fig. 2A). Within the QUOD retrieval biopsy cohort, only BulkFormer-TabPFN using absolute and maximum aggregation produced models with C-statistics whose 95% confidence intervals excluded 0.5. Consistent with the NMP findings, models omitting the BulkFormer step demonstrated no evidence of predictive discrimination.

**Fig. 4:**
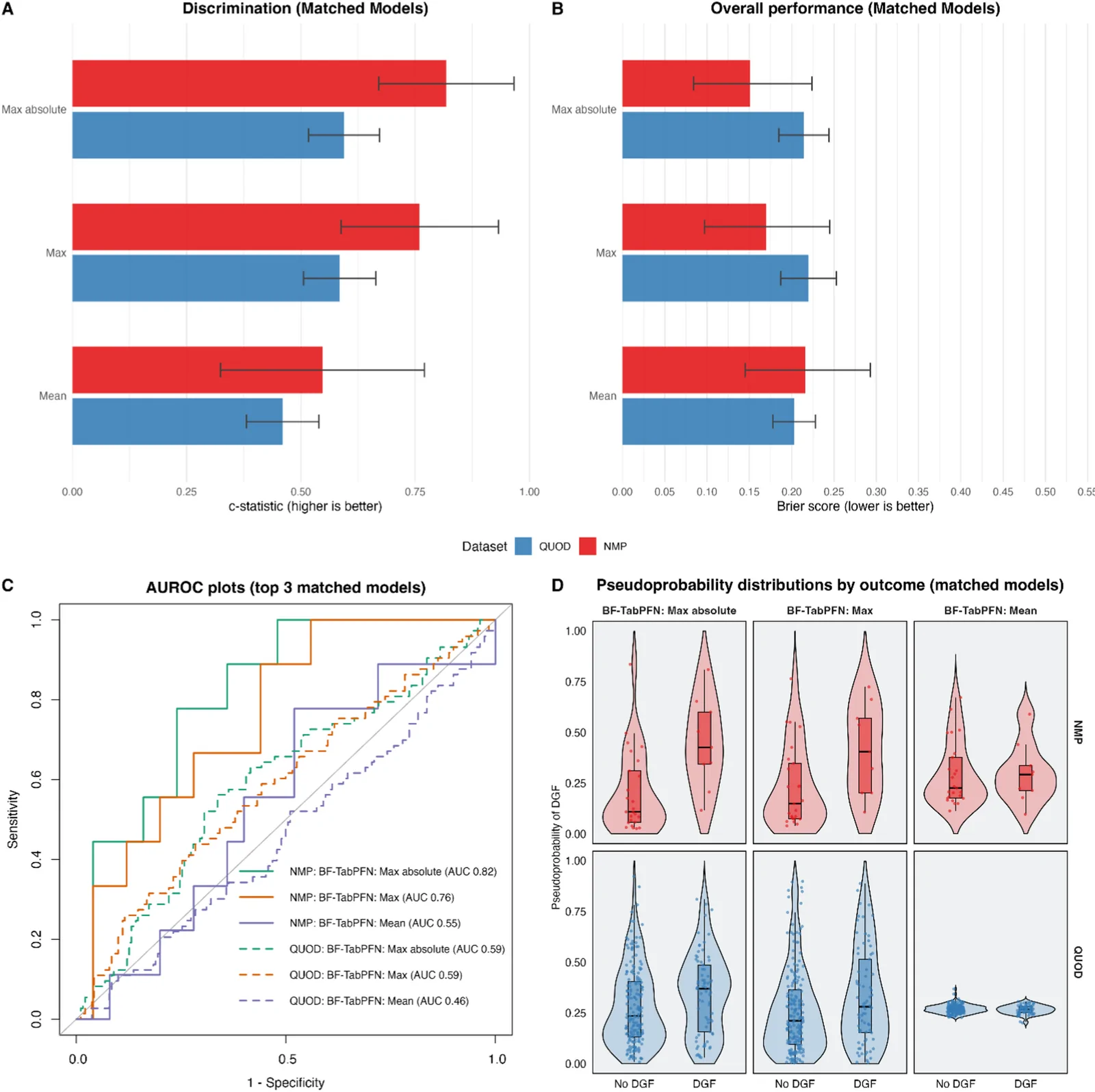
Comparing model pipeline predictive performance in the normothermic machine perfusion cohort versus the cold storage cohort (QUOD retrieval biopsy). The discrimination (measured using c-statistic) and overall performance (Brier score) for BulkFormer-TabPFN models are shown in panel (**A**) and (**B**), labelled with the BulkFormer gene-level embedding aggregation method. Predictive performance in the NMP and QUOD cohort are represented by red and blue bars respectively. Error bars represent 95% confidence intervals. (**C**) Receiver-operating characteristic curves for BulkFormer-TabPFN models, stratified by study cohort. (**D**) Distribution of predicted probabilities stratified by subsequent delayed graft function status, with facets for aggregation method and study cohort (red = NMP, blue = QUOD).

With an identical model pipeline, outcome definition, and an identical transcriptomic pipeline performed in the same laboratory^6,20^, predictive performance was significantly higher when using NMP biopsies versus the QUOD kidney biopsies (Fig. 4). For example, with the maximum absolute aggregation approach, discrimination was strong in the NMP cohort (c-statistic = 0.82, 95% CI  0.67–0.97) but substantially lower in the QUOD cohort (c-statistic = 0.59, 95% CI  0.52–0.67).

## Discussion

We show for the first time the ability of an AI pipeline combining BulkFormer Version 2 and TabPFN v2.5 to generate outcome predictions from raw transcriptomic data. In the setting of the small datasets examined here, the addition of BulkFormer for context-aware distillation of gene data into embeddings significantly improved predictive performance, compared with pipelines using TabPFN alone. Predictive performance was sensitive to the method used to aggregate gene-level into sample-level embeddings; we found that maximum absolute aggregation of BulkFormer embeddings was optimal, a method which we believe has not been described previously^14,15^. All code is freely available on GitHub and includes tools enabling readers to apply the pipeline to their own raw count data^18^.

Due to the small sample size, focus on a single short-term outcome, and single centre collection/processing of samples, this work is exploratory and is clearly not a clinical prediction model which is ready for deployment. Nevertheless, there are important messages and opportunities for transplantation. Firstly, this adds to the body of evidence for improved organ assessment using NMP^2–4^; with our pipeline, transcriptomic signatures during NMP led to superior predictions compared with transcriptomic signatures at retrieval. Importantly, the NMP trial and QUOD retrieval biopsy cohorts were analysed completely independently, with no sharing of samples, features, or contextual information at any stage. QUOD cohort predictions were not informed by the NMP cohort, and the observed differences in performance are due to the underlying biological signal themselves. We believe improved prediction with NMP biopsies reflects sampling after both cold ischaemia and the early phase of reperfusion injury, when the biological consequences of injury may be more transcriptionally apparent.

Secondly, we have provided evidence that these predictions are responsive to the delivery of a novel NMP therapy (Cytokine filters)^6^. With larger cohorts of outcome-linked biopsy data, this could be a potential surrogate endpoint in preclinical or early-phase trials, which could potentially reduce the need for larger, more expensive clinical trials in the future. As a continuous variable it offers increased power and lower sample sizes in comparison to binary endpoints, and it is intrinsically tied to post-transplant outcome. This is particularly important given the substantial volume of preclinical NMP therapeutic studies, where robust, translatable efficacy readouts are currently limited^34,35^. Although larger cohorts of outcome-linked data are required, the code we provide^18^ acts as a blueprint for the generation of DGF predicted probabilities from kidney biopsy raw count data.

Transcriptomic assessment of kidney biopsies has entered select clinical practice for post-transplant biopsies, most notably with the Molecular Microscope Diagnostics System (MMDx)^36^. Although sequencing costs have reduced substantially (<$150 for bulk RNA-Seq), turn-around time remains a hurdle in the time-pressured setting of organ assessment. Advances in next-generation sequencing with nanopore have already been reported to provide sample-to-answer times of just 6 h^37^. The analysis of the resulting raw count data with BulkFormer-TabPFN will only take seconds to minutes, as it requires a single forward pass through the pipeline. As RNA-Seq workflows continue to improve, and technologies for extending organ preservation time evolve^38^, transcriptomic assessment could one day guide organ accepting decisions^38–41^. To achieve this aim, predictions should focus on patient-centred, longer-term outcomes that are more relevant to clinical decision-making than transient delayed graft function alone.

This manuscript provides a blueprint for how AI foundation models can be combined to make rapid predictions from raw count data, without manual bioinformatic analysis. A key advantage of our pipeline is that it performs predictions ‘zero-shot’, meaning that no model retraining is performed, and none of the model weights are changed. This is possible as both BulkFormer and TabPFN are foundation models. They use transformer-based architectures, and train on massive amounts of data^12,14,15^. After this training, they can then take new labelled data as a ‘prompt’ and provide predictions with a single forward pass through the network, akin to how a large language model can take text as a prompt and provide a response (without retraining the model weights).

This contrasts with standard approaches to generate predictions from RNA-Seq data. Standard statistical methods generally rely on feature selection (selection of ‘significant’ genes) prior to subsequent modelling. This process is inherently unstable, and in small datasets overfitting and overoptimism are virtually guaranteed^9–11^. These issues are magnified if variable/feature selection is performed using association with the outcome being predicted^42,43^. Even options for dimensionality reduction such as principal components can distort inference by inflating type I error, whilst losing biologically relevant signal^44,45^. Established machine-learning methods, including random forests and gradient boosting, remain vulnerable to the combined challenges of high feature dimensionality, noise, and small sample size, often resulting in unstable models which are overfitted to noise^46,47^. Direct benchmarking against methods that require model fitting is therefore challenging, as differences in the training paradigm, the data, and the validation design would be confounded with the approach itself.

TabPFN is trained to approximate Bayesian inference, and has demonstrated state-of-the-art performance for tabular data predictions across a wide range of disciplines, outperforming bespoke models using established machine learning approaches^13^. In addition to improved predictive performance, TabPFN offers practical advantages: prediction is rapid as model retraining is entirely avoided, implementation is often simpler as hyperparameter tuning is not required, and performance is particularly strong in the setting of low sample-size^12^. One limitation of TabPFN is its significantly decreased performance with data that exceeds 2000 dimensions^13,17^. This is clearly a challenge for transcriptomic data, where the number of genes exceeds 10,000–20,000.

This is where we turned to BulkFormer version 2^27^, which utilises a graph neural-network to incorporate known gene–gene interactions, and a performer-based architecture which was trained on over 500,000 bulk RNA-Seq samples^14,15^. It takes RNA-Seq data on 20,010 genes, and distils this into a compressed set of embeddings which represent biological signal, whilst minimising information loss. In the original BulkFormer manuscript these embeddings were fed into a random forest model to generate predictions^14,15^. BulkFormer outperformed existing single-cell trained models on a range of tasks including patient prognosis prediction^14,15^.

The benefits of these two models are complementary for transcriptomic data. TabPFN performs well across multiple disciplines, especially in the setting of small sample sizes, however struggles with high feature count data. BulkFormer allows the number of features to be reduced, whilst preserving biological signal. Our findings support these conceptual benefits. TabPFN would also allow very simple integration of donor characteristics into the same model, as additional features, which may further improve predictions.

Despite these advantages, and growing use of both models individually, to our knowledge only a single pre-print article has described the combination of BulkFormer and TabPFN^48^. This found no benefit of integrating BulkFormer with TabPFN. There are several potential reasons for the difference in conclusions with this study. Firstly, they aggregated gene-level embeddings using mean aggregation only; an approach which also failed to improve predictions in our manuscript. They also used older versions of both BulkFormer and TabPFN, and used datasets with over 10,000 samples (which is not where TabPFN shows the most benefit)^13,27,48^.

Transcriptomic prediction of allograft outcomes has also been approached through curated multi-organ atlases. Robertson et al. assembled transcriptomic data across four transplanted organs and predicted DGF and other allograft pathologies, with performance improving when data from multiple organs was pooled^49^. Their approach required the outcome to be used twice during model development. First to identify the top 500 genes associated with clinical outcomes, including DGF, and again to train the prediction model itself on pre-implantation biopsies. Notably, models trained on multi-organ data consistently outperformed those trained on single organ data. While their work addresses the same clinical challenge as ours, the prediction of DGF from pre-implantation kidney biopsies, our approach differs sufficiently to make direct comparison difficult. Our proposed pipeline relies entirely on single organ data, and its models have never been trained on the outcome of interest. No genes are selected by outcome association, no coefficients are estimated, and the encoder which compresses the 20,010 genes has no exposure to transplant outcomes. Taking these differences into account, our best performing pipeline achieved a c-statistic of 0.82 in NMP biopsies, using kidney data alone. Their best reported AUC for DGF was 0.89, but this was achieved using a model trained on pooled kidney and liver data rather than kidney data alone. No equivalent kidney-only result was reported, precluding a more direct comparison of AUC results.

In contrast to the conventional structure of model development and external validation described above, our study falls outside of the standard paradigm of internal and external validation of clinical prediction models, as it uses zero-shot models^50^. As there is no model retraining, many of the standard approaches for internal validation studies, such as optimism calculation/correction, do not apply. However, this does not represent true external validation, as the study cohort used as the input is still used for generating predictions^50,51^. The most recent TRIPOD + AI guidelines explicitly excluded foundational models, and the TRIPOD-LLM guidelines do not cover tabular models. In the future new guidelines will be required to cover the application of foundational tabular models for clinical prediction^50^.

The main limitations relate to the retrospective nature of the study, and reliance on available sample numbers and metadata. Overall, there was a small available sample size of biopsy data linked to post-transplant outcome, with minimal metadata on other important clinical factors (such as donor age). As a result, it is unclear how much this transcriptomic data adds to standard clinical factors, and we were unable to account for important donor/recipient factors such as age or pre-emptive transplants. However, our BulkFormer-TabPFN pipeline achieved superior predictive discrimination (c-statistic) than DGF predictions models using clinical data, and predictions were modifiable by treatment (in kidney pairs with identical clinical variables)^52^. Further research using larger more richly labelled datasets is also required to explore the ability to predict clinical outcomes beyond transient early function.

Whilst combining BulkFormer-TabPFN improved performance compared with TabPFN alone, combining two models amplifies the “black-box” nature of foundation models, making it challenging to attribute predictions to specific genes. Future work is needed to understand the biological processes captured by individual BulkFormer embeddings and to develop more explainable approaches for interpreting TabPFN predictions.

Another limitation is that all biopsies from all cohorts were processed by the same laboratory and subjected to an identical RNA-Seq generation pipeline, which may limit generalisability and transferability to external cohorts. Although external validation does not apply to zero-shot approaches in the same way as it does to models that require retraining, evaluating performance on independently processed datasets remains essential to establish generalisability. This will be an important area for future research as additional transcriptomic NMP datasets become available. In addition, NMP biopsies linked to outcome data were only available at the 1-hour timepoint. As transcriptomic profiles evolve over the course of NMP^6^, the applicability of models derived from early biopsies to samples obtained after prolonged perfusion remains uncertain and warrants further investigation. The benefit of our approach is that as larger outcome-linked RNA-Seq datasets become available, they can be incorporated into our pipeline, allowing iterative evaluation of generalisability, consistency, transportability and predictive performance.

In conclusion, transcriptomic data poses a unique challenge due to the high number of genes, relative to the often small sample size. Standard methods for prediction rely on feature selection, which leads to overfitting and optimism about model performance. Here we combine two state of the art artificial intelligence models, BulkFormer and TabPFN. These transformer-based foundation models can generate predictions “zero-shot”, without retraining on new data. This manuscript is the first to demonstrate the synergistic benefits of combining these models in terms of predictive performance. Our freely available code offers a blueprint to apply this pipeline to generate predictions from raw RNA-Seq, which could be applied in a wide range of contexts beyond deceased donor kidney transplantation.

## Supplementary Information

Below is the link to the electronic supplementary material.

Supplementary Material 1

## Data Availability

All data and code are freely available open access via our GitHub repository for the project https://github.com/TxDataIncubator/BulkFormer_with_TabPFN_RNA-Seq_Kidney_Transcriptomics.

## References

[1] Olawade, D. B., Marinze, S., Qureshi, N., Weerasinghe, K. & Teke, J. Transforming organ donation and transplantation: Strategies for increasing donor participation and system efficiency. *Eur. J. Intern. Med.* **133**, 14–24. 10.1016/j.ejim.2024.11.010 (2025). 39572291

[2] Jaynes, C. L., Goggins, W. C., Holzner, M. L., Garonzik-Wang, J. & Leuvenink, H. G. D. No Kidney left behind: Rescuing unused donor kidneys for transplant at the first centralized assessment and repair center. *Transpl. Int.* **38**, 15424. 10.3389/ti.2025.15424 (2025). PMC12698490 41394518

[3] Hosgood, S. A., Barlow, A. D., Hunter, J. P. & Nicholson, M. L. Ex vivo normothermic perfusion for quality assessment of marginal donor kidney transplants. *Br. J. Surg.* **102** (11), 1433–1440. 10.1002/bjs.9894 (2015). 26313559

[4] Tingle, S. J. et al. Contrast-enhanced ultrasound to assess kidney quality during ex situ normothermic machine perfusion. *Transpl. Int.* **38**, 14268. 10.3389/ti.2025.14268 (2025). PMC11999844 40242325

[5] Hosgood, S. A. et al. Normothermic machine perfusion versus static cold storage in donation after circulatory death kidney transplantation: a randomized controlled trial. *Nat. Med.* **29** (6), 1511–1519. 10.1038/s41591-023-02376-7 (2023). PMC10287561 37231075

[6] Ferdinand, J. R. et al. Cytokine absorption during human kidney perfusion reduces delayed graft function-associated inflammatory gene signature. *Am. J. Transpl.* **21** (6), 2188–2199. 10.1111/ajt.16371 (2021). PMC8246774 33098231

[7] Spiers, H. V. M. et al. Transcriptomic profiling during normothermic machine perfusion of human kidneys reveals a pro-inflammatory cellular landscape and gene expression signature associated with severe ischemia-reperfusion injury and delayed graft function. *Front. Immunol.* **16** 10.3389/fimmu.2025.1679251 (2025). PMC12586191 41200199

[8] Rao, P. S. et al. A comprehensive risk quantification score for deceased donor kidneys: the kidney donor risk index. *Transplantation* **88** (2), 231–236. 10.1097/TP.0b013e3181ac620b (2009). 19623019

[9] Clarke, R. et al. The properties of high-dimensional data spaces: implications for exploring gene and protein expression data. *Nat. Rev. Cancer*. **8** (1), 37–49. 10.1038/nrc2294 (2008). PMC2238676 18097463

[10] Simon, R., Radmacher, M. D., Dobbin, K. & McShane, L. M. Pitfalls in the use of DNA microarray data for diagnostic and prognostic classification. *J. Natl. Cancer Inst.* **95** (1), 14–18. 10.1093/jnci/95.1.14 (2003). 12509396

[11] Ambroise, C. & McLachlan, G. J. Selection bias in gene extraction on the basis of microarray gene-expression data. *Proc. Natl. Acad. Sci.* **99** (10), 6562–6566. 10.1073/pnas.102102699 (2002). PMC124442 11983868

[12] Hollmann, N. et al. Accurate predictions on small data with a tabular foundation model. *Nature* **637** (8045), 319–326. 10.1038/s41586-024-08328-6 (2025). PMC11711098 39780007

[13] Grinsztajn, L. et al. TabPFN-2.5: Advancing the state of the art in tabular foundation models. *arXiv*. Preprint posted online February 5, 2026:arXiv:2511.08667. 10.48550/arXiv.2511.08667

[14] Kang, B., Fan, R., Yi, M., Cui, C. & Cui, Q. A large-scale foundation model for bulk transcriptomes. *bioRxiv*. Preprint posted online June 17, 2025:2025.06.11.659222. 10.1101/2025.06.11.659222

[15] Kang, B., Fan, R., Yi, M., Cui, C. & Cui, Q. BulkFormer: A large-scale foundation model for bulk transcriptomes. *Cels* **17** (7). 10.1016/j.cels.2026.101657 (2026). 42385705

[16] Zhang, Q., Tan, Y. S., Tian, Q. & Li, P. TabPFN: One Model to Rule Them All? *arXiv*. Preprint posted online November 27, 2025:arXiv:2505.20003. 10.48550/arXiv.2505.20003

[17] Ye, H. J., Liu, S. Y. & Chao, W. L. A closer look at TabPFN v2: Understanding its strengths and extending its capabilities. *arXiv*. Preprint posted online June 11, 2025:arXiv:2502.17361. 10.48550/arXiv.2502.17361

[18] Tingle, S. J. & Kourounis, G. TxDataIncubator/BulkFormer_with_TabPFN_RNA-Seq_Kidney_Transcriptomics: Data and code repository for project. GitHub. Accessed February 27, (2026). https://github.com/TxDataIncubator/BulkFormer_with_TabPFN_RNA-Seq_Kidney_Transcriptomics

[19] Kourounis, G. & Tingle, S. TxDataIncubator/BulkFormer_with_TabPFN_RNA-Seq_Kidney_Transcriptomics: Zenodo v1.0.0. Published online 2026.

[20] Zhang, R. et al. Assessment of biological organ age using molecular pathology in pre-transplant kidney biopsies. *Kidney Int.* **106** (2), 302–316. 10.1016/j.kint.2024.03.028 (2024). 38692408

[21] Venkataraman, K., Irish, G. L., Collins, M. G. & Clayton, P. A. The association between early graft function, donor type and long-term kidney transplant outcomes. *Transpl. Int.* **38**, 14197. 10.3389/ti.2025.14197 (2025). PMC12122319 40454295

[22] Dyer, S. C. et al. Ensembl 2025. *Nucleic Acids Res.* **53** (D1), D948–D957. 10.1093/nar/gkae1071 (2025). PMC11701638 39656687

[23] Durinck, S. et al. BioMart and bioconductor: a powerful link between biological databases and microarray data analysis. *Bioinformatics* **21** (16), 3439–3440. 10.1093/bioinformatics/bti525 (2005). 16082012

[24] Durinck, S., Spellman, P. T., Birney, E. & Huber, W. Mapping identifiers for the integration of genomic datasets with the R/Bioconductor package biomaRt. *Nat. Protoc.* **4** (8), 1184–1191. 10.1038/nprot.2009.97 (2009). PMC3159387 19617889

[25] Braschi, B. et al. Genenames.org: the HGNC and VGNC resources in 2019. *Nucleic Acids Res.* **47** (D1), D786–D792. 10.1093/nar/gky930 (2019). PMC6324057 30304474

[26] Kang, B. KangBoming/BulkFormer. Published online February 7, 2026. https://github.com/KangBoming/BulkFormer (accessed 10 Jan 2026).

[27] Kang, B., Fan, R., Cui, C. & Cui, Q. A large-scale foundation model for human bulk transcriptomes (version 2). Published online Dec. 11. 10.5281/zenodo.15744294 (2025).

[28] Prior-Labs/tabpfn_2_5 · Hugging Face. November 11, 2025. https://huggingface.co/Prior-Labs/tabpfn_2_5 (accessed 27 Feb 2026).

[29] Love, M. I., Huber, W. & Anders, S. Moderated estimation of fold change and dispersion for RNA-seq data with DESeq2. *Genome Biol.* **15** (12), 550. 10.1186/s13059-014-0550-8 (2014). PMC4302049 25516281

[30] Ritchie, M. E. et al. limma powers differential expression analyses for RNA-sequencing and microarray studies. *Nucleic Acids Res.* **43** (7), e47. 10.1093/nar/gkv007 (2015). PMC4402510 25605792

[31] Calster, B. V. et al. Evaluation of performance measures in predictive artificial intelligence models to support medical decisions: overview and guidance. *Lancet Digit. Health*. **7** (12). 10.1016/j.landig.2025.100916 (2025). 41391983

[32] Robin, X. et al. pROC: an open-source package for R and S + to analyze and compare ROC curves. *BMC Bioinform.* **12**, 77 (2011). 10.1186/1471-2105-12-77 PMC3068975 21414208

[33] benvancalster. benvancalster/PerfMeasuresOverview. Published online February 5. (2026). https://github.com/benvancalster/PerfMeasuresOverview (accessed 27 Feb 2026).

[34] Franchin, B. et al. Normothermic kidney perfusion: Current status and future perspectives. *Kidney Int. Rep.* **10** (9), 2943–2952. 10.1016/j.ekir.2025.06.041 (2025). PMC12446984 40980641

[35] Rabelink, T. J. et al. Opportunities and challenges with the implementation of normothermic machine perfusion in kidney transplantation. *Nat. Commun.* **16** (1), 6883. 10.1038/s41467-025-60410-3 (2025). PMC12296735 40715058

[36] Halloran, P. F., Madill-Thomsen, K. S. & Reeve, J. The molecular phenotype of kidney transplants: Insights from the MMDx project. *Transplantation* **108** (1), 45–71. 10.1097/TP.0000000000004624 (2024). PMC10718223 37310258

[37] Gu, W. et al. Rapid pathogen detection by metagenomic next-generation sequencing of infected body fluids. *Nat. Med.* **27** (1), 115–124. 10.1038/s41591-020-1105-z (2021). PMC9020267 33169017

[38] Dumbill, R. et al. Prolonged normothermic perfusion of the kidney prior to transplantation: a historically controlled, phase 1 cohort study. *Nat. Commun.* **16** (1), 4584. 10.1038/s41467-025-59829-5 (2025). PMC12085653 40382321

[39] Saju, A. F., Mukundan, A., Divyashree, M. S., Hariharapura, R. C. & Mahadev Rao, A. RNA diagnostics and therapeutics: a comprehensive review. *RNA Biol.* **22** (1), 1–11. 10.1080/15476286.2024.2449277 (2025). PMC12713360 39744758

[40] Chu, V. T. et al. Implementation and outcomes of a rapid response genomic hospital epidemiology programme at an academic medical centre over 7 years. *Lancet Microbe*. **7** (2), 101277. 10.1016/j.lanmic.2025.101277 (2026). PMC13108401 41512899

[41] Shuja, A. Advances in RNA-Seq data analysis: Towards more reliable clinical translation: Advances in RNA-Seq data analysis: Towards reliable clinical translation. *Dev. Medico-Life-Sci.* **2**(8), 1–2. 10.69750/dmls.02.08.0143 (2025).

[42] Collins, G. S. et al. Evaluation of clinical prediction models (part 1): from development to external validation. *BMJ* **384**, e074819. 10.1136/bmj-2023-074819 (2024). PMC10772854 38191193

[43] Harrell, F. E. xRegression modeling strategies: with applications, to linear models, logistic and ordinal regression, and survival analysis, 2nd ed. Heidelberg: Springer. *Biometrics*. **72**(3), 1006–1007. 10.1111/biom.12569 (2016).

[44] Jolliffe, I. T. & Cadima, J. Principal component analysis: a review and recent developments. *Philos. Trans. Math. Phys. Eng. Sci.* **374** (2065), 20150202. 10.1098/rsta.2015.0202 (2016). PMC4792409 26953178

[45] Nygaard, V., Rødland, E. A. & Hovig, E. Methods that remove batch effects while retaining group differences may lead to exaggerated confidence in downstream analyses. *Biostatistics* **17** (1), 29–39. 10.1093/biostatistics/kxv027 (2016). PMC4679072 26272994

[46] Feldner-Busztin, D. et al. Dealing with dimensionality: the application of machine learning to multi-omics data. *Bioinformatics* **39** (2), btad021. 10.1093/bioinformatics/btad021 (2023). PMC9907220 36637211

[47] Kumar, S., Agarwal, A. & Chatterjee, S. Feature learning augmented with sampling and heuristics (FLASH) improves model performance and biomarker identification. *NPJ Syst. Biol. Appl.* **11**, 137. 10.1038/s41540-025-00614-x (2025). PMC12700939 41387731

[48] Zhou, S., Agarwal, V., Gopinath, A. & Kassis, T. The limitations of TabPFN for high-dimensional RNA-seq analysis. *bioRxiv*. Preprint posted online September 17, 2025:2025.08.15.670537. 10.1101/2025.08.15.670537

[49] Robertson, H. et al. Decoding the hallmarks of allograft dysfunction with a comprehensive pan-organ transcriptomic atlas. *Nat. Med.* **30** (12), 3748–3757. 10.1038/s41591-024-03030-6 (2024). PMC11645273 38890530

[50] Collins, G. S. et al. TRIPOD + AI statement: updated guidance for reporting clinical prediction models that use regression or machine learning methods. *BMJ* **385**, e078378. 10.1136/bmj-2023-078378 (2024). PMC11019967 38626948

[51] Gallifant, J. et al. The TRIPOD-LLM reporting guideline for studies using large language models. *Nat. Med.* **31** (1), 60–69. 10.1038/s41591-024-03425-5 (2025). PMC12104976 39779929

[52] Irish, W. D., Ilsley, J. N., Schnitzler, M. A., Feng, S. & Brennan, D. C. A risk prediction model for delayed graft function in the current era of deceased donor renal transplantation. *Am. J. Transplant.* **10** (10), 2279–2286. 10.1111/j.1600-6143.2010.03179.x (2010). 20883559

