# Supplementary material for: Combining BulkFormer and TabPFN to predict post-transplant function from kidney biopsies during normothermic machine perfusion or cold storage
Source: Sci Rep. 2026 Aug 27;16:36430. doi: 10.1038/s41598-026-68541-3 (PMC13727666; doi:10.1038/s41598-026-68541-3)
Supplement: Supplementary file 1 [file 41598_2026_68541_MOESM1_ESM.pdf]

# Supplementary Figures

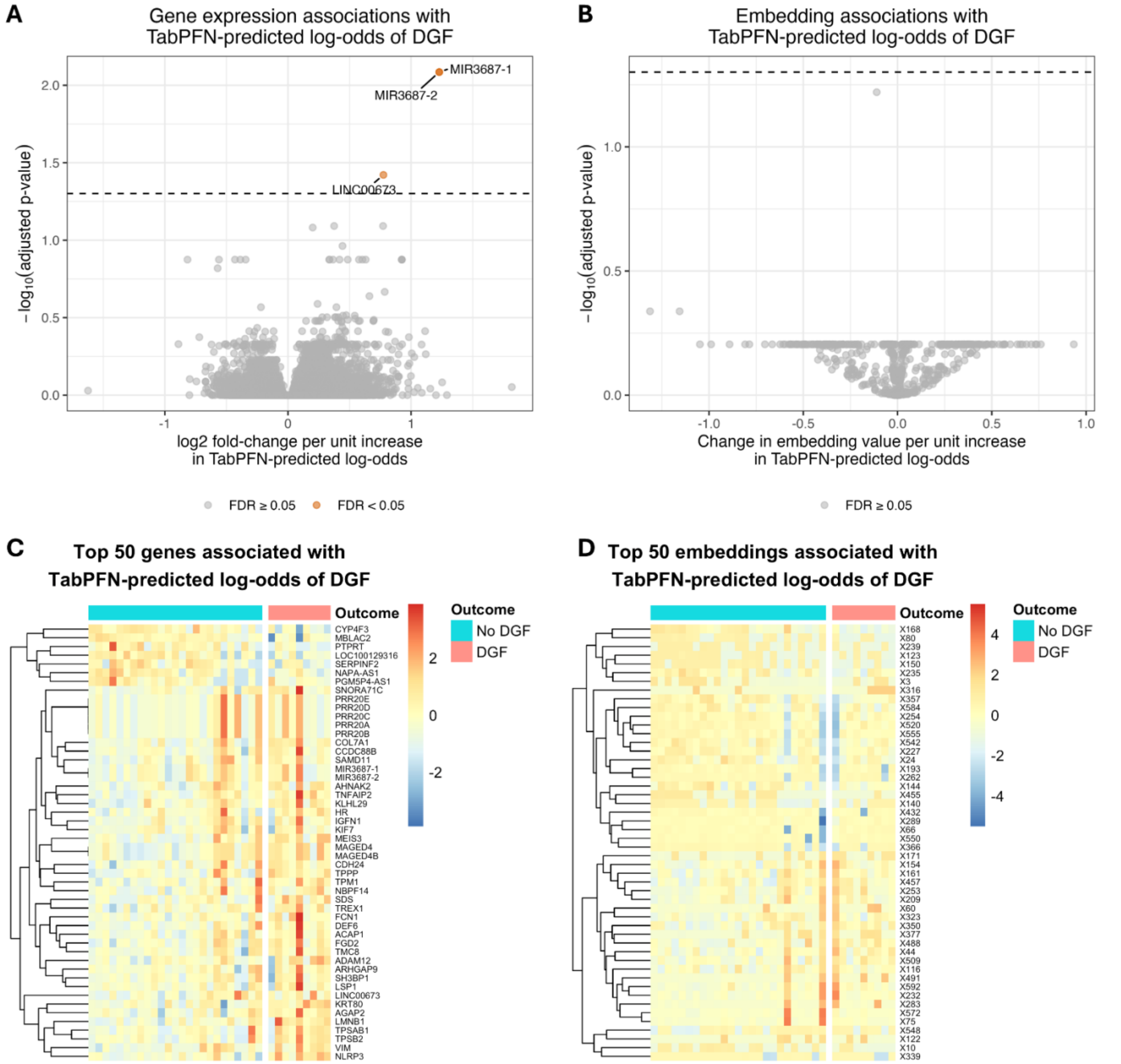


Supplementary Figure 1 – Associations between transcriptomic/BulkFormer features and TabPFN-predicted risk of delayed graft function (DGF). A&B) Volcano plots showing the associations between transcriptomic features and TabPFN-predicted log-odds of DGF. Gene expression was analysed using DESeq2 negative-binomial regression (A), and BulkFormer embeddings were analysed using limma linear models with empirical Bayes moderation (B). Dashed lines indicate a Benjamini–Hochberg false discovery rate of 0.05. C&D) Heatmaps show the 50 genes and embedding dimensions with the lowest P values in the corresponding DESeq2 and limma models, representing the features most strongly associated with TabPFN predictions. Gene expression values were variance-stabilised before visualisation, and both gene and embedding values were scaled by row. Samples were ordered by observed DGF outcome. Overall, no individual gene or BulkFormer embedding showed a uniformly consistent pattern across samples.

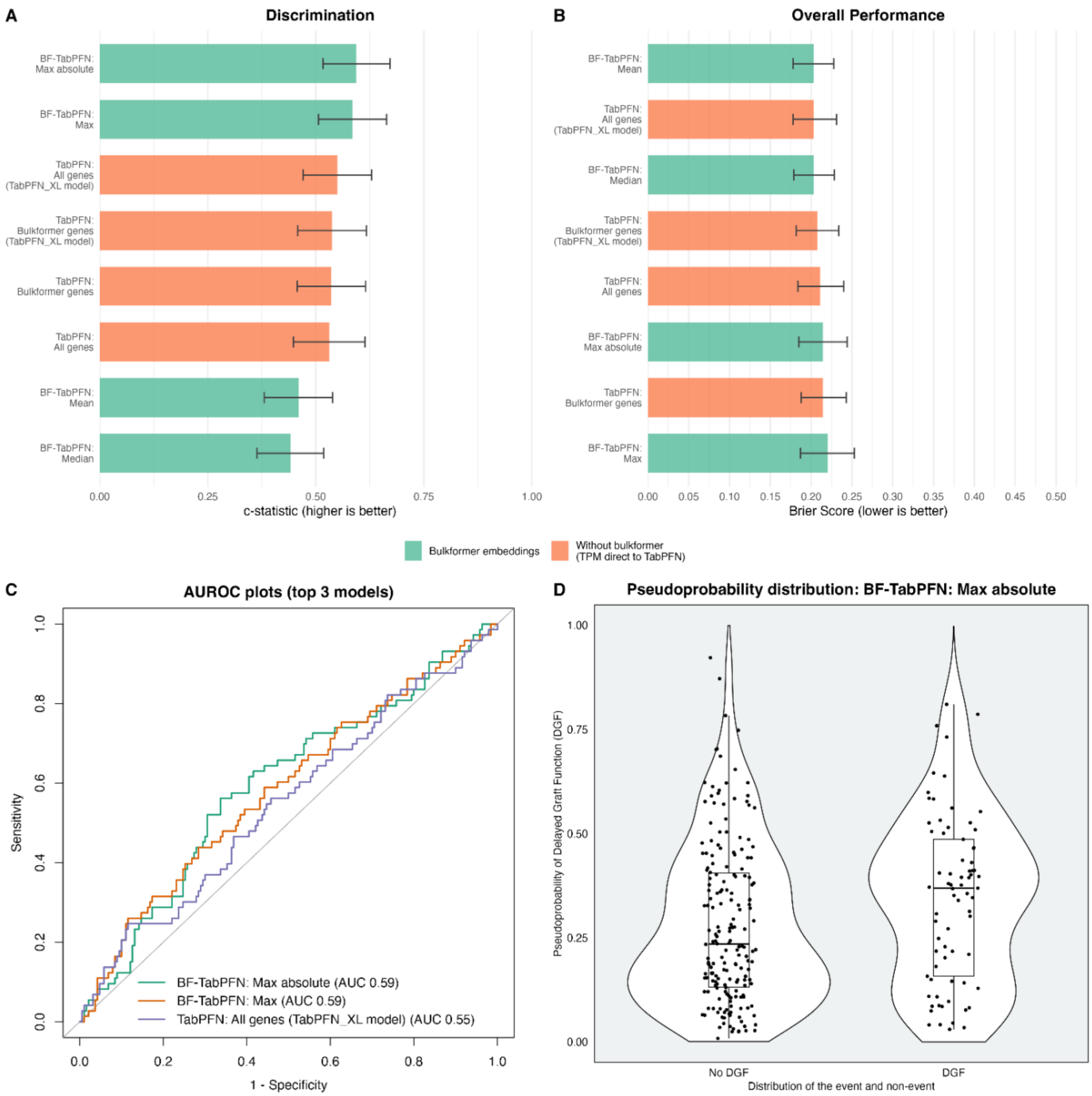


Supplementary Figure 2 – Predictive performance for early post-transplant kidney function (defined as Delayed Graft Function; DGF) in the QUOD retrieval biopsy cohort. The discrimination (measured using c-statistic) and overall performance (Brier score) for each of the tested models are shown in panel A and B, and are ranked best to worst. Models using the full BulkFormer-TabPFN pipeline are shown in green, and are labelled with which BulkFormer method was used for aggregation of gene-level embeddings (e.g. Max absolute). Models using only TabPFN directly on the transcriptomic data (without BulkFormer) are shown in orange, and labelled with which genes were used, and whether the TabPFN default or high feature count (XL model) were used. C) Receiver-operating characteristic curves for the top three performing models by c-statistic. D) Distribution of predicted probabilities stratified by subsequent delayed graft function status.

# Supplementary tables

Supplementary table 1 – All model performance metrics for all models in the NMP and QUOD cohorts. For models using the BulkFormer-TabPFN pipeline (BF-TabPFN) the labels reflect the BulkFormer method for aggregating gene-level embeddings. For models using the just TabPFN on the raw transcript per million data (without BulkFormer), the label represents which genes were used, and whether the TabPFN default or high feature count (XL model) were used.

| Cohort | n | Model label | c-statistic (95% CI) | AUPRC (95% CI) | AP (95% CI) | Brier (95% CI) | Log loss (95% CI) | Scaled Brier (95% CI) | McFadden $R^2$ (95% CI) | Cox-Snell $R^2$ (95% CI) | Nagelkerke $R^2$ (95% CI) |
|---|---|---|---|---|---|---|---|---|---|---|---|
| NMP | 34 | BF-TabPFN: Max absolute | 0.82 (0.67, 0.97) | 0.52 (0.23, 0.90) | 0.58 (0.30, 0.91) | 0.15 (0.08, 0.22) | 15.46 (9.77, 21.72) | 0.22 (-0.25, 0.49) | 0.21 (-0.14, 0.44) | 0.22 (-0.15, 0.41) | 0.32 (-0.23, 0.59) |
| NMP | 34 | BF-TabPFN: Max | 0.76 (0.59, 0.93) | 0.46 (0.21, 0.84) | 0.52 (0.28, 0.85) | 0.17 (0.10, 0.24) | 17.10 (11.29, 23.43) | 0.13 (-0.34, 0.40) | 0.13 (-0.22, 0.35) | 0.14 (-0.25, 0.34) | 0.20 (-0.40, 0.49) |
| NMP | 34 | BF-TabPFN: Mean | 0.55 (0.32, 0.77) | 0.27 (0.12, 0.56) | 0.31 (0.17, 0.61) | 0.22 (0.14, 0.29) | 21.35 (15.62, 27.80) | -0.11 (-0.55, 0.10) | -0.09 (-0.39, 0.09) | -0.10 (-0.48, 0.10) | -0.15 (-0.74, 0.14) |
| NMP | 34 | BF-TabPFN: Max (99.9) | 0.54 (0.32, 0.77) | 0.27 (0.12, 0.56) | 0.31 (0.16, 0.60) | 0.25 (0.15, 0.36) | 26.14 (16.11, 37.58) | -0.29 (-1.04, 0.10) | -0.33 (-0.92, 0.05) | -0.47 (-1.68, 0.06) | -0.68 (-2.49, 0.08) |
| NMP | 34 | BF-TabPFN: Median | 0.53 (0.30, 0.76) | 0.27 (0.12, 0.57) | 0.32 (0.15, 0.61) | 0.21 (0.15, 0.29) | 21.31 (15.55, 27.65) | -0.11 (-0.58, 0.10) | -0.09 (-0.41, 0.09) | -0.10 (-0.45, 0.09) | -0.15 (-0.72, 0.14) |
| NMP | 34 | BF-TabPFN: Max absolute (99.9) | 0.49 (0.24, 0.75) | 0.28 (0.11, 0.62) | 0.33 (0.14, 0.65) | 0.23 (0.15, 0.34) | 25.66 (16.11, 38.34) | -0.21 (-0.84, 0.12) | -0.31 (-0.86, 0.07) | -0.42 (-1.70, 0.07) | -0.62 (-2.45, 0.11) |
| NMP | 34 | TabPFN: All genes | 0.43 (0.17, 0.69) | 0.26 (0.10, 0.61) | 0.31 (0.14, 0.63) | 0.22 (0.14, 0.30) | 22.17 (15.79, 28.83) | -0.14 (-0.57, 0.07) | -0.13 (-0.44, 0.04) | -0.16 (-0.52, 0.04) | -0.23 (-0.82, 0.07) |
| NMP | 34 | TabPFN: Bulkformer genes | 0.42 (0.15, 0.69) | 0.25 (0.10, 0.58) | 0.30 (0.14, 0.61) | 0.23 (0.15, 0.31) | 23.20 (15.97, 30.67) | -0.16 (-0.56, 0.07) | -0.18 (-0.49, 0.03) | -0.23 (-0.68, 0.04) | -0.34 (-1.02, 0.05) |
| NMP | 34 | TabPFN: All genes (TabPFN_XL model) | 0.39 (0.13, 0.65) | 0.23 (0.09, 0.55) | 0.28 (0.12, 0.59) | 0.22 (0.15, 0.30) | 22.32 (16.51, 28.68) | -0.15 (-0.53, 0.03) | -0.14 (-0.41, 0.02) | -0.17 (-0.48, 0.02) | -0.25 (-0.79, 0.03) |
| NMP | 34 | TabPFN: Bulkformer genes (TabPFN_XL model) | 0.38 (0.12, 0.63) | 0.22 (0.09, 0.53) | 0.27 (0.12, 0.56) | 0.23 (0.15, 0.31) | 23.09 (16.66, 30.74) | -0.16 (-0.49, -0.00) | -0.17 (-0.46, -0.01) | -0.22 (-0.63, -0.01) | -0.33 (-0.96, -0.02) |
| QUOD | 263 | BF-TabPFN: Max absolute (99.9) | 0.60 (0.51, 0.68) | 0.43 (0.32, 0.55) | 0.44 (0.34, 0.56) | 0.20 (0.18, 0.23) | 162.91 (144.07, 184.85) | -0.01 (-0.13, 0.09) | -0.05 (-0.16, 0.05) | -0.06 (-0.20, 0.05) | -0.09 (-0.30, 0.08) |
| QUOD | 263 | BF-TabPFN: Max (99.9) | 0.59 (0.52, 0.67) | 0.39 (0.29, 0.50) | 0.40 (0.30, 0.51) | 0.20 (0.18, 0.23) | 165.19 (142.99, 187.78) | -0.02 (-0.13, 0.07) | -0.06 (-0.18, 0.03) | -0.08 (-0.23, 0.04) | -0.11 (-0.33, 0.06) |

| Cohort | n | Model label | c-statistic (95% CI) | AUPRC (95% CI) | AP (95% CI) | Brier (95% CI) | Log loss (95% CI) | Scaled Brier (95% CI) | McFadden R² (95% CI) | Cox-Snell R² (95% CI) | Nagelkerke R² (95% CI) |
|---|---|---|---|---|---|---|---|---|---|---|---|
| QUOD | 263 | BF-TabPFN: Max absolute | 0.59 (0.52, 0.67) | 0.34 (0.26, 0.45) | 0.35 (0.27, 0.47) | 0.21 (0.18, 0.24) | 168.62 (147.29, 190.09) | -0.07 (-0.19, 0.04) | -0.09 (-0.20, 0.02) | -0.11 (-0.26, 0.03) | -0.15 (-0.37, 0.04) |
| QUOD | 263 | BF-TabPFN: Max | 0.58 (0.51, 0.66) | 0.34 (0.26, 0.45) | 0.35 (0.27, 0.47) | 0.22 (0.19, 0.25) | 178.86 (152.96, 207.86) | -0.10 (-0.23, 0.02) | -0.15 (-0.29, -0.02) | -0.20 (-0.42, -0.02) | -0.28 (-0.60, -0.03) |
| QUOD | 263 | TabPFN: All genes (TabPFN_XL model) | 0.55 (0.47, 0.63) | 0.33 (0.24, 0.44) | 0.34 (0.26, 0.45) | 0.20 (0.18, 0.23) | 157.77 (142.34, 175.44) | -0.01 (-0.08, 0.04) | -0.02 (-0.07, 0.03) | -0.02 (-0.09, 0.03) | -0.03 (-0.13, 0.05) |
| QUOD | 263 | TabPFN: Bulkformer genes (TabPFN_XL model) | 0.54 (0.46, 0.62) | 0.31 (0.23, 0.40) | 0.31 (0.24, 0.41) | 0.21 (0.18, 0.23) | 160.72 (144.87, 176.38) | -0.04 (-0.10, 0.02) | -0.04 (-0.09, 0.02) | -0.04 (-0.11, 0.02) | -0.06 (-0.16, 0.03) |
| QUOD | 263 | TabPFN: Bulkformer genes | 0.54 (0.46, 0.61) | 0.30 (0.23, 0.39) | 0.31 (0.24, 0.40) | 0.21 (0.19, 0.24) | 166.33 (148.36, 185.07) | -0.07 (-0.15, 0.01) | -0.07 (-0.15, -0.00) | -0.09 (-0.20, -0.00) | -0.13 (-0.28, -0.00) |
| QUOD | 263 | TabPFN: All genes | 0.53 (0.45, 0.61) | 0.33 (0.25, 0.45) | 0.34 (0.27, 0.46) | 0.21 (0.18, 0.24) | 165.08 (146.42, 184.60) | -0.05 (-0.14, 0.03) | -0.06 (-0.14, 0.01) | -0.08 (-0.18, 0.01) | -0.11 (-0.27, 0.01) |
| QUOD | 263 | BF-TabPFN: Mean | 0.46 (0.38, 0.54) | 0.25 (0.19, 0.33) | 0.26 (0.20, 0.34) | 0.20 (0.18, 0.23) | 156.84 (142.61, 171.23) | -0.01 (-0.03, -0.00) | -0.01 (-0.03, -0.00) | -0.01 (-0.04, -0.00) | -0.02 (-0.05, -0.00) |
| QUOD | 263 | BF-TabPFN: Median | 0.44 (0.36, 0.52) | 0.24 (0.19, 0.30) | 0.24 (0.20, 0.31) | 0.20 (0.18, 0.23) | 157.22 (143.05, 171.51) | -0.01 (-0.04, -0.01) | -0.01 (-0.03, -0.00) | -0.01 (-0.04, -0.01) | -0.02 (-0.05, -0.01) |
